# An algorithm-based meta-analysis of genome- and proteome-wide data identifies a combination of potential plasma biomarkers for colorectal cancer

**DOI:** 10.1038/s41598-019-51999-9

**Published:** 2019-10-30

**Authors:** Danuta R. Gawel, Eun Jung Lee, Xinxiu Li, Sandra Lilja, Andreas Matussek, Samuel Schäfer, Renate Slind Olsen, Margaretha Stenmarker, Huan Zhang, Mikael Benson

**Affiliations:** 1 Centre for Personalized Medicine, Linköping University, Linköping, Sweden; 2 Department of Otorhinolaryngology, Yonsei University College of Medicine, Seoul, Korea; 3 Laboratory Medicine, Division of Psychiatrics & Rehabilitation & Diagnostics, Region Jönköping County, Jönköping, Sweden; 4 Division of Clinical Microbiology, Department of Laboratory Medicine, Karolinska Institutet, Karolinska University Hospital Huddinge, Stockholm, Sweden; 5 Karolinska University Laboratory, Karolinska University Hospital, Solna, Sweden; 6 Pathology Laboratory, Division of Psychiatrics & Rehabilitation & Diagnostics, Region Jönköping County, Jönköping, Sweden; 7 Center for Translational Microbiome Research, Department of Microbiology, Tumor and Cell Biology, Karolinska Institutet, Stockholm, Sweden; 8 Department of Paediatrics, Jönköping, Region Jönköping County, and Department of Clinical and Experimental Medicine, Linköping University, Linköping, Sweden

**Keywords:** Cytokines, Diagnostic markers

## Abstract

Screening programs for colorectal cancer (CRC) often rely on detection of blood in stools, which is unspecific and leads to a large number of colonoscopies of healthy subjects. Painstaking research has led to the identification of a large number of different types of biomarkers, few of which are in general clinical use. Here, we searched for highly accurate combinations of biomarkers by meta-analyses of genome- and proteome-wide data from CRC tumors. We focused on secreted proteins identified by the Human Protein Atlas and used our recently described algorithms to find optimal combinations of proteins. We identified nine proteins, three of which had been previously identified as potential biomarkers for CRC, namely CEACAM5, LCN2 and TRIM28. The remaining proteins were PLOD1, MAD1L1, P4HA1, GNS, C12orf10 and P3H1. We analyzed these proteins in plasma from 80 patients with newly diagnosed CRC and 80 healthy controls. A combination of four of these proteins, TRIM28, PLOD1, CEACAM5 and P4HA1, separated a training set consisting of 90% patients and 90% of the controls with high accuracy, which was verified in a test set consisting of the remaining 10%. Further studies are warranted to test our algorithms and proteins for early CRC diagnosis.

## Introduction

Colorectal cancer (CRC) is the third most common form of cancer. Globally, it affects more than 1.2 million individuals each year, and causes some 700,000 deaths^1,2^.

Survival rates have improved over the last 30 years due to better diagnostics, surgical and oncological treatment. Survival is, however, still most related to the tumor stage at diagnosis. At stages I, the 5-year survival rate is 87–92% and in stage II it’s 49–87%, for stage III 53–89%, and at Stage IV 11–12%^3,4^ (https://www.cancer.org/cancer/colon-rectal-cancer/detection-diagnosis-staging/survival-rates.html). It is therefore essential to find the tumor as early as possible. This has led to recommendations for national screening programs in many countries^5^. Those programs are mainly based on detecting occult blood in faeces, followed by colonoscopy. This has increased early detection of CRC and reduced mortality. A problem, however, is that screening programs will result in many healthy subjects being subjected to unnecessary colonoscopy. This investigation is costly and perceived as unpleasant or painful by many subjects. Furthermore, it’s associated with a small risk of perforation. Thus, there is the need for improved and more accurate biomarker for early diagnosis of CRC. Several such biomarkers have been proposed, including assays for multiple proteins, microRNAs, DNA methylation, or tumor DNA in blood or stools^6–12^.

However, in general these tests are not used in clinical settings for different reasons, such as costs, complexity of measurement or that their accuracy need confirmation in larger studies. One important observation of such studies is that combinations of biomarkers are superior to individual ones^13^. This suggests that strategies to ideally, identify a limited number of highly accurate biomarkers that can be measured in routine clinical settings, are needed. However, the scale of the identification problem is indicated by a recent survey of medical literature, which identified 383 proteins, 94 mRNAs, 35 DNAs and 185 other forms of potential biomarkers for CRC^14^.

Here, we propose a strategy for identification of potential protein biomarkers for CRC that can be measured in blood, which is based on meta-analysis of published genome- and proteome- wide analyses of CRC tumor tissues and adjacent tissues (AT)^15–18^. We focused on differentially expressed genes whose protein products were potentially released extracellularly, according to the Human Protein Atlas^19^. In order to identify optimal combinations of a limited number of proteins, we used our recently described classification algorithm^20^. Finally, we tested those biomarkers in plasma from patients with newly diagnosed CRC and healthy controls.

## Results

### Biomarker selection

**Figure 1:**
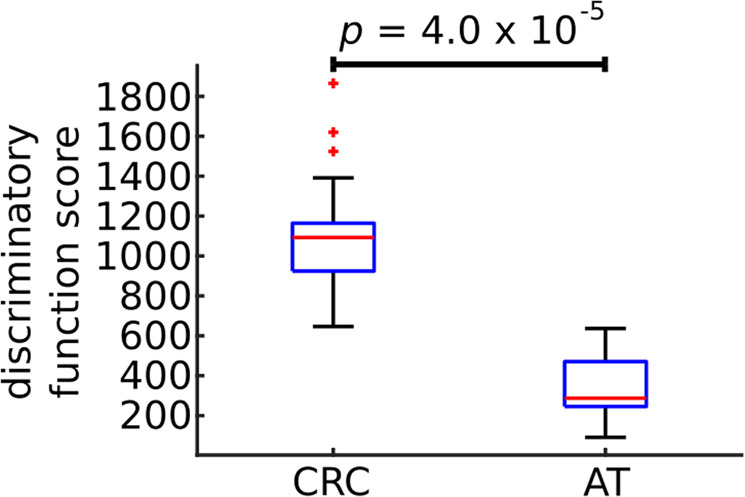
Classification accuracy of 22 paired samples – colorectal cancer tumor samples (CRC) and adjacent tissue (AT). Boxplot presents discriminatory function score for CRC and AT groups (sum of the unit of parts per million [ppm] reported in^15^ for selected proteins: PLOD1, P4HA1, LCN2, GNS, C12orf10, P3H1, TRIM28, CEACAM5, MAD1L1. *p* value was calculated using double-sided Wilcoxon Signed Rank test. The bars in the boxes represent median, 25^th^ and 75^th^ percentiles, while whiskers extend to ±2.7 σ.

In order to prioritize proteins as putative biomarkers of CRC we analyzed publically available proteome profiling of CRC samples and paired AT samples collected from 22 subjects^15^. First, we preselected proteins that were (a) differentially expressed (corrected for multiple testing paired t-test *p* value < 0.01); (b) upregulated in CRC samples compared to AT samples (fold change >2); and (c) predicted to be secreted by the Human Protein Atlas. We identified 113 such proteins. Using random elastic net (Materials and Methods) we prioritized proteins based on their predictive value to discriminate CRC from AT (Supplementary Data 1). For further analyses we selected the top nine proteins: PLOD1 (Q02809), P4HA1 (P13674), LCN2 (P80188), GNS (P15586), C12orf10 (Q9HB07), P3H1 (Q32P28), TRIM28 (Q13263), CEACAM5 (P06731), MAD1L1 (Q9Y6D9); (randomized elastic net frequency >0.45; Supplementary Data 1; Materials and Methods). We found that the sum of the expression values of those nine proteins discriminated CRC and AT with high accuracy (Area Under receiver operating characteristic Curve AUC = 1, Wilcoxon Signed Rank test *p* = 4.0 × 10^−5^; Fig. 1). This was significantly higher than when using nine random proteins (permutation test *p* < 1.0 × 10^−4^, OR = 1.66). Out of those nine proteins, three were reported before as possible CRC biomarkers; namely CEACAM5 (commonly known as CEA), LCN2 and TRIM28^16^.

### Biomarker tests in independent proteomic datasets

**Figure 2:**
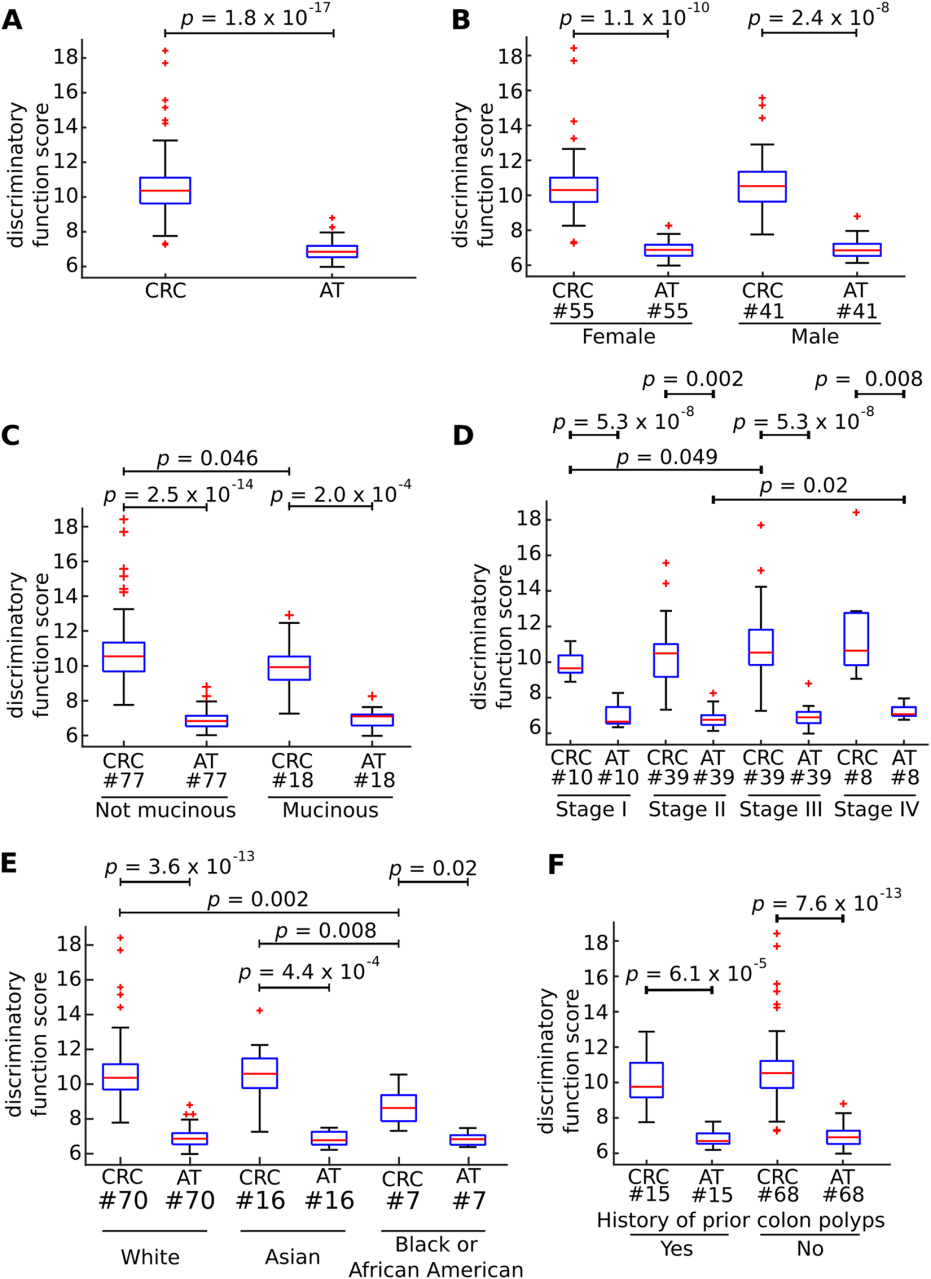
Classification accuracy of 96-paired samples – colorectal cancer tumor samples (CRC) and adjacent tissue (AT). Boxplot presenting discriminatory function score for CRC and AT groups (sum of the two to the power of Unshared Log Ratio scores reported in the study for selected proteins: PLOD1, P4HA1, LCN2, GNS, C12orf10, P3H1, TRIM28, CEACAM5, MAD1L1). Significant *p* values were calculated using double-sided Wilcoxon Signed Rank test for paired samples and Wilcoxon Rank Sum test for unpaired samples. The bars in the boxes represent median, 25^th^ and 75^th^ percentiles, while whiskers extend to ±2.7 σ. Numbers below the boxplots denote number of observations per category. (**A**) Discrimination between samples derived from tumor (CRC) and AT. (**B**) Discrimination between CRC and AT samples in males and females separately. (**C**) Discrimination between CRC and AT samples depending on histological subtype. (**D**) Discrimination between CRC and AT samples depending on the tumor stage. (**E**) Discrimination between CRC and AT samples depending on race. (**F**) Discrimination between CRC and AT samples depending on prior colon polyp history.

In order to assess the reproducibility of our results we analyzed two independent publicly available proteome profiling datasets of CRC. First, we analyzed a dataset consisting of 101 individuals – that data were generated by National Cancer Institute Clinical Proteomic Tumor Analysis Consortium (CPTAC). The data consists of 96 paired samples obtained from tumor site (CRC) and AT. Secondly, we validated that the selected nine proteins could separate tumor from AT. We obtained nearly perfect classification accuracy (AUC = 0.99, Wilcoxon Signed Rank test *p* = 1.8 × 10^−17^, Fig. 2A), which was higher than expected by chance for randomly selected nine proteins (permutation test *p* = 1.0 × 10^−4^, OR = 2.21). In the same study, authors report clinical data including sex, race, histological subtype, history of prior colon polyps. We therefore tested if any of those covariates had an impact on the sample classification (Fig. 2B–F, Supplementary Figs 1, 2 and Supplementary Data 2). We found significant differences of the discriminatory function score only between mucinous and non-mucinous tumors (Wilcoxon Rank Sum test *p* = 0.046; Fig. 2C); pathological tumor stage I and III (double sided Wilcoxon Rank sum test *p* = 0.049; Fig. 2D); AT samples from patients with tumor stage II and IV (Wilcoxon Rank sum test *p* = 0.02, Fig. 2D); between races - black or African American patients versus Asian and versus White, *p* value = 0.008, and 0.002 respectively, Fig. 2E); expression or no-expression of MHL1 and PMS2 (Wilcoxon Rank Sum test *p* = 0.02, Supplementary Fig. 1E; and *p* = 0.008, Supplementary Fig. 2B respectively, Supplementary Data 2). Furthermore, we tested the correlation between pathological tumor stage and discriminatory function score and found no significant correlation (Pearson PCC = 0.12, *p* = 0.12). However, as shown in Fig. 2 those covariates did not have a significant impact on overall classification accuracy.

**Figure 3:**
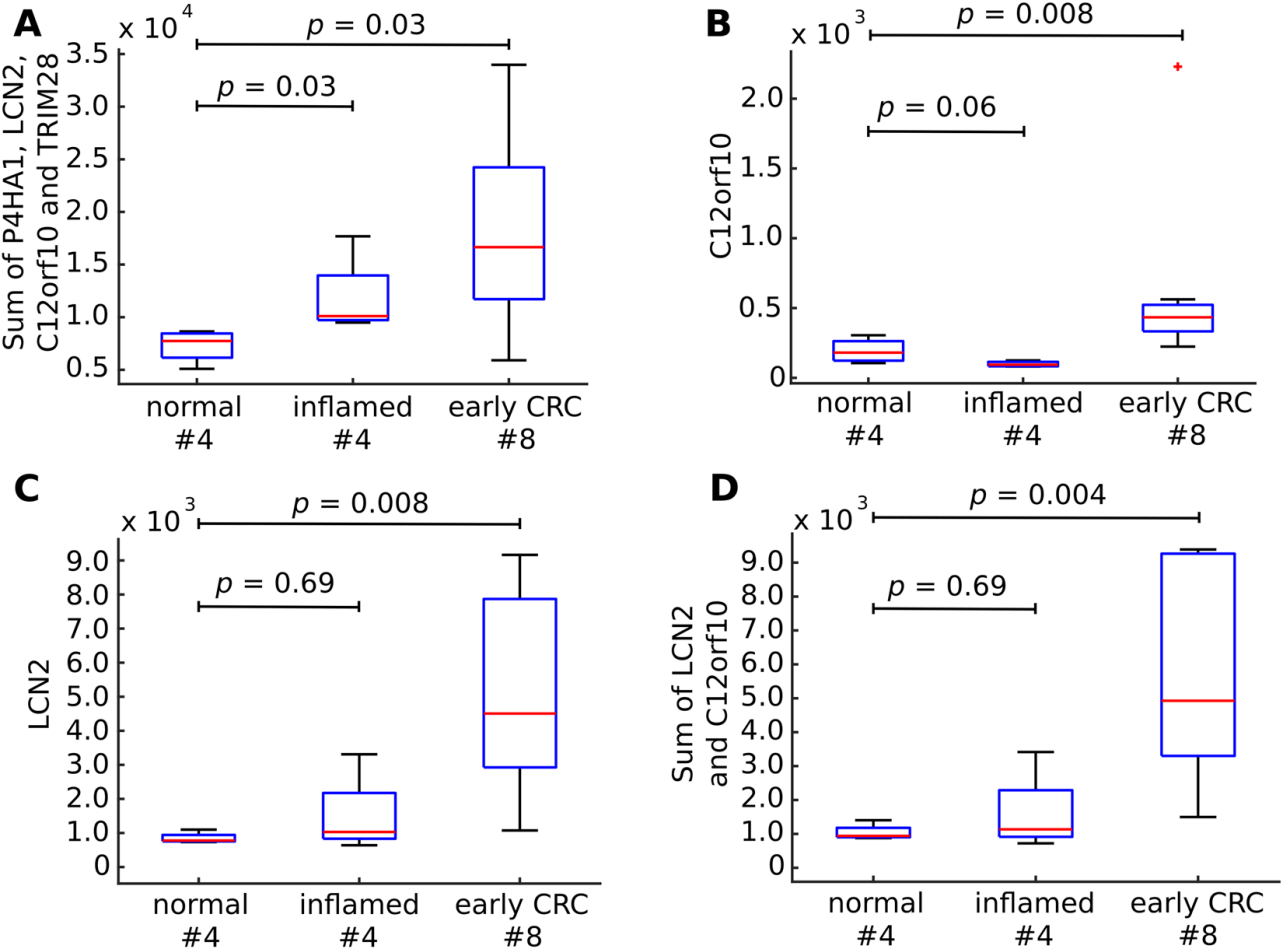
Classification accuracy of 16 samples – early colorectal cancer tumor samples (CRC), normal and inflamed tissue. (**A**) Boxplot presenting discriminatory function score (sum of the four of nine present in the dataset proteins: P4HA1, LCN2, C12orf10, TRIM28). (**B**) C12orf10 expression differences in normal, inflamed and early CRC tissues. (**C**) LCN2 expression differences in normal, inflamed and early CRC tissues. (**D**) Sum of LCN2 and C12orf10 proteins separates normal and early CRC tissue samples; Significance *p* value was calculated using double-sided Wilcoxon Rank Sum test. The bars in the boxes represent median, 25^th^ and 75^th^ percentiles, while whiskers extend to ±2.7 σ. Numbers below the boxplots denote number of observations per category.

Encouraged by those results we tested yet another proteomic dataset consisting of 76 tissue samples, in which four to five patient sample digests were pooled. In total, proteomics analyses were performed on eight pools from colorectal tissue samples obtained from early stages of CRC, eight pools of apparently normal tissue (at surgical margin) samples and four pools of inflamed mucosa samples^17^. This dataset contains only four out of nine selected putative biomarkers: P4HA1 (P13674), LCN2 (P80188), C12orf10 (Q9HB07) and TRIM28 (Q13263). For this reason, we created a new classifier based on the sum of those four tentative CRC biomarkers, which yielded a high classification accuracy for discriminating early CRC from normal tissue (AUC = 0.91, Wilcoxon Rank Sum test, unpaired samples, *p* = 0.03, Fig. 3A). We also found a significant difference between normal and inflamed tissue (Wilcoxon Rank Sum test *p* = 0.03, Fig. 3A). However, the combination of the four proteins did not yield higher AUC score than expected by chance for random four proteins (*p* = 0.08, OR = 1.68). Therefore, we asked if individual proteins could discriminate normal from tumor tissues. Indeed, we found that LCN2 and C12orf10 differentiated between those two conditions (AUC = 0.97, Wilcoxon Rank Sum test *p* = 0.008 for both proteins), which was higher than expected by chance for a random single gene classifier (permutation test *p* = 0.03, OR = 1.86 for both proteins; Fig. 3B,C). A combination of those two proteins gave even higher classification accuracy (AUC = 1, Wilcoxon Rank Sum test *p* = 0.004; Fig. 3D), which is higher than expected for two random proteins (permutation test *p* = 1.0 × 10^−4^, OR = 1.9). This suggested that a subset of the nine proteins might be sufficient for a highly accurate classification of patients and controls.

### Biomarker tests in independent transcriptomic datasets

**Figure 4:**
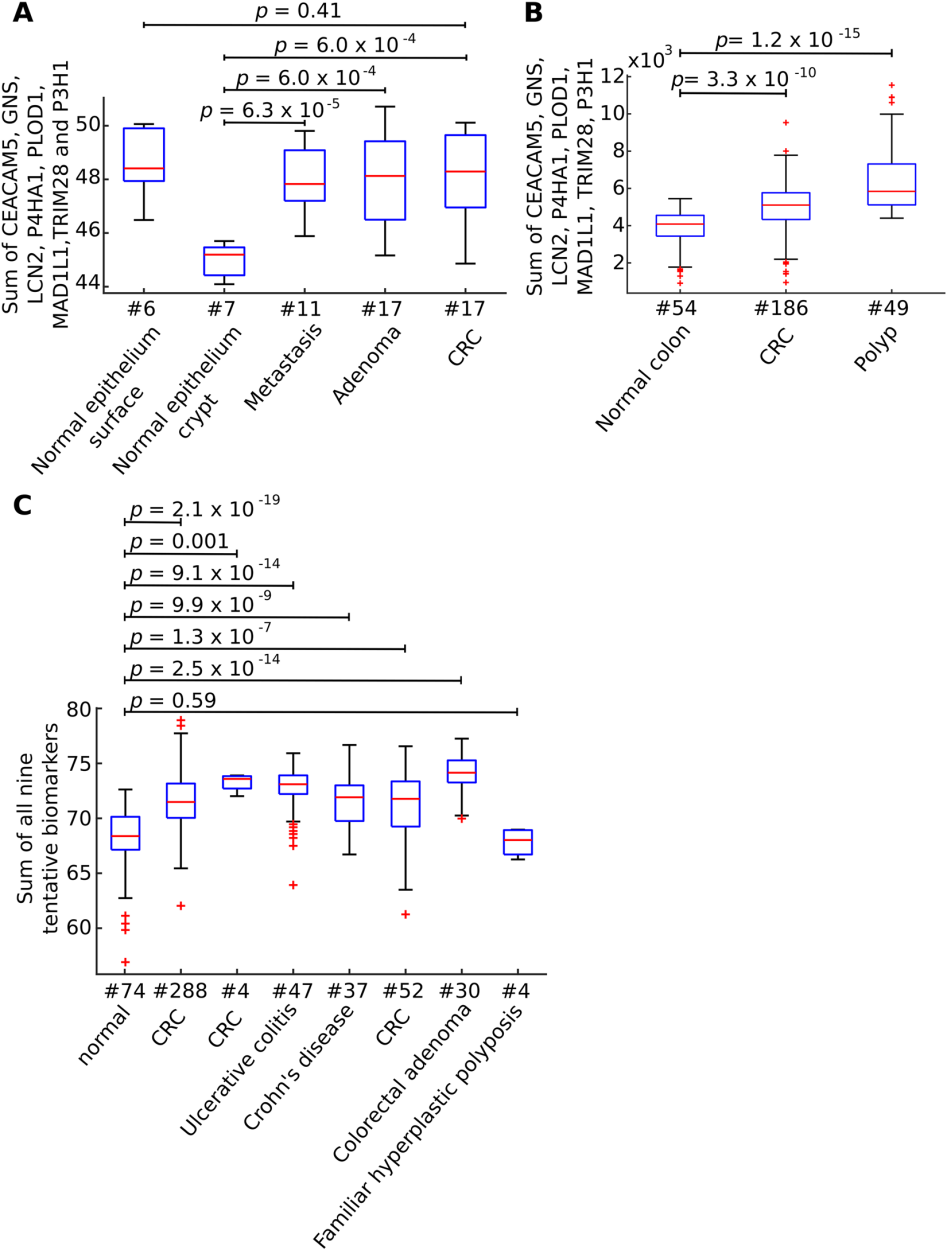
Classification accuracy of colorectal tumour (CRC) samples and adjacent tissue (AT) in transcriptomic profiling datasets. (**A**) Transcriptome profiling of colorectal samples from 6 normal surface epithelium, 7 normal crypt epithelium, 17 CRC, 11 metastasis, 17 adenomas (in total 19 subjects). (**B**) 54 normal colon tissue, 186 CRC and 49 polyps (**C**) 74 normal samples, CRC samples from three different studies (n = 4, 288 and 52, respectively), 30 adenomas, 4 familial hyperplastic polyposis, 47 ulcerative colitis, 37 Crohn’s disease. For each plot *p* values were calculated using double-sided Wilcoxon Rank Sum test. The bars in the boxes represent median, 25^th^ and 75^th^ percentiles, while whiskers extend to ±2.7 σ.

We also tested 3 transcriptome profiling studies of CRC. In case some of the selected nine genes were not expressed in the tested dataset, classifiers were built using genes that were expressed. Firstly, we analyzed a dataset consisting of 6 normal surface epithelia, 7 normal crypt epithelia, 17 CRC, 11 metastases, 17 adenoma samples (in total 19 subjects; EGEOD-77955). In this dataset *C12orf10* gene expression was missing. Therefore, we created a classifier based on the remaining eight genes. We obtained a high classification accuracy when comparing normal crypt epithelium samples to CRC, metastases and adenoma samples (AUC >0.95, Wilcoxon Rank Sum test *p* < 6.1 × 10^−4^; Fig. 4A; Table 1). However, in comparison to normal surface samples we didn’t get good separation of the groups (AUC <0.43, Wilcoxon Rank Sum test *p* > 0.25; Fig. 4A; Table 1).

**Table 1:** Classification area under the receiver operating characteristic^37^ of 6 normal surface epithelium and 7 normal crypt epithelium versus 17 CRC, 11 metastasis, and 17 adenoma samples, respectively.

	Metastasis	Adenoma	CRC
**versus normal surface epithelium**			
AUC	0.32	0.42	0.41
*p*	0.26	0.60	0.55
**versus normal crypt epithelium**			
AUC	1.00	0.96	0.96
*p*	6.3 × 10^−5^	6.0 × 10^−4^	6.0 × 10^−4^

Significance was calculated using double-sided Wilcoxon Rank Sum test (*p*).

Similarly, in another transcriptome profiling study tested (E-GEOD-41258) we obtained good separation between groups (AUC >0.77, Wilcoxon Rank Sum test *p* < 3.4 × 10^−10^; Fig. 4B; Table 2). Here, we compared 54 normal colon tissue samples with 186 primary CRC and 49 polyp samples. This dataset also lacked expression profiles of *C12orf10*, and therefore the classifier was made using the remaining eight genes.

**Table 2:** Classification’s area under the receiver operating characteristic^37^ of 54 normal colon tissues versus 186 primary CRC, 49 polyp samples, respectively.

	Primary CRC	Polyp
AUC	0.78	0.96
*P*	3.3 × 10^−10^	1.2 × 10^−15^

Significance calculated using double-sided Wilcoxon Rank Sum test was reported (*p*).

Finally, we analyzed a dataset (E-MTAB-3732) that aggregated and normalized microarray data from colorectal samples from 74 healthy tissues, three studies of CRC (n = 4, 288 and 52 respectively), 4 familial hyperplastic polyposis, 30 colorectal adenomas, 47 ulcerative colitis, 37 Crohn’s disease. All nine tentative biomarkers were profiled. In this dataset, we compared normal colorectal tissue samples to other groups. This yielded high separation for most comparisons (AUC >0.82; Wilcoxon Rank Sum test *p* < 0.002; Fig. 4C; Table 3). We also found significant differences between normal tissue compared to CRC (moderate separation AUC = 0.78, Wilcoxon Rank Sum test *p* = 1.3 × 10^−7^). However, the classifier didn’t differentiate between normal colorectal tissue and familial hyperplastic polyposis (AUC = 0.42, Wilcoxon Rank Sum test *p* = 0.59).

**Table 3:** Classification area under the receiver operating characteristic^37^ of 74 normal samples versus 37 Crohn’s disease, three CRC studies (n = 4 colon tumour, 288 colorectal adenocarcinoma and 52 colorectal carcinoma, respectively), 30 colorectal adenoma, 4 familiar hyperplastic polyposis and 47 ulcerative colitis.

	Crohn’s disease	CRC (study 1)	CRC (study 2)	Colorectal adenoma	CRC (study 3)	Familial hyperplastic polyposis	Ulcerative colitis
AUC	0.83	0.99	0.84	0.98	0.78	0.42	0.90
*p*	9.9 × 10^−9^	0.001	2.1 × 10^−19^	2.5 × 10^−14^	1.3 × 10^−7^	0.59	9.1 × 10^−14^

Significance was calculated using double-sided Wilcoxon Rank Sum test (*p*).

In summary, analyses of transcriptome profiling data supported that the chosen nine tentative CRC biomarkers could separate colorectal tumor samples from apparently normal samples. The pathogenic relevance of the identified biomarkers was further supported by network analysis, which showed that they were highly interconnected and functionally related (Supplementary Fig. 3).

### Analyses of nine potential protein biomarkers in plasma samples from CRC patients and healthy controls

**Figure 5:**
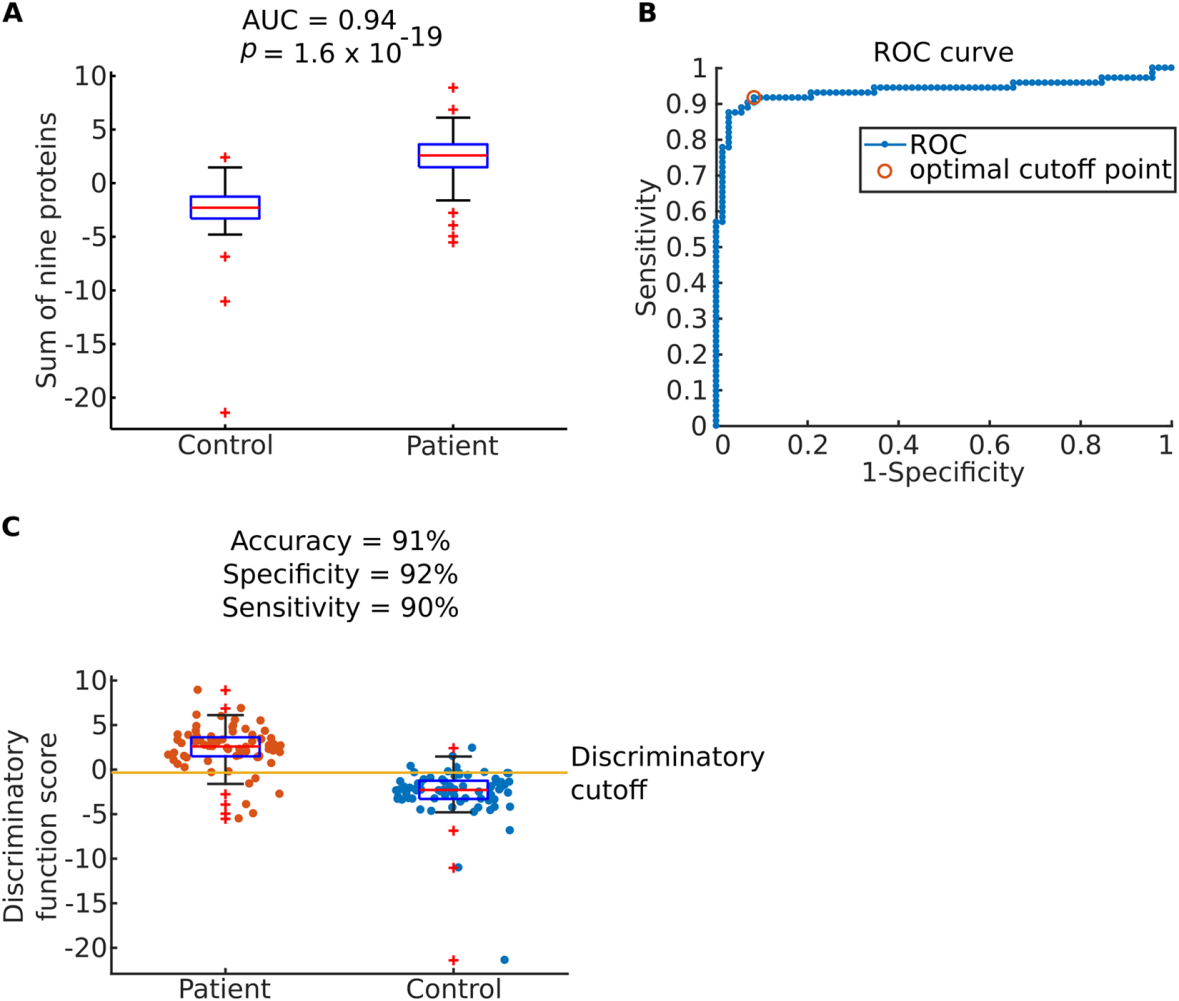
Classification accuracy of 72 patients and 72 control samples in training group. (**A**) Boxplot presenting discriminatory function score based on the protein expression in plasma samples measured with ELISA. Discriminatory function score was calculated as sum of the nine proteins. The bars in the boxes represent median, 25^th^ and 75^th^ percentiles, while whiskers extend to ±2.7 σ. (**B**) Receiver operating characteristic curve^36^ obtained for classifier based on the sum of nine proteins measures in plasma samples of patients and controls with ELISA. Optimal operating point (discriminatory function cut-off value) is marked with red circle, and it corresponds to the score value of −0.34. (**C**) Boxplot showing discriminatory function score for patients and controls in training set. The selected discriminatory function cut-off value is presented with solid, vertical, yellow line. Each dot represents individual sample. The bars in the boxes represent median, 25^th^ and 75^th^ percentiles, while whiskers extend to ±2.7 σ.

We proceeded to test all nine selected biomarkers in plasma samples obtained from 80 patients with CRC and 80 controls. We found that seven of the proteins differed significantly between patients and controls: PLOD1, median 10 (0–10) vs. 0.19 (0–7.9) ng/mL, *p* = 1.19 × 10^−21^, LCN2, 2.0 (0–10) vs. 2.3 (1.3–4.2) ng/mL, *p* = 4.84 × 10^−2^, MAD1L1, 0 (0–3.1) vs. 0 (0–10) ng/mL, *p* = 2.73 × 10^−2^, CEACAM5, 0 (0–0.45) vs. 0 (0–3.8) pg/mL, *p* = 1.52 × 10^−3^, P4HA1, 3.7 (0–17 vs. 5.9 (1.4–22) ng/mL, *p* = 4.88 × 10^−6^, TRIM28, 0 (0–0.82) vs. 1.14 (0–20) ng/mL, *p* = 1.00 × 10^−27^ and GNS, 7.1 (2.8–13) vs. 10 (5.3–14) ng/mL, *p* = 1.05 × 10^−14^. By contrast, no significant differences were found for C12orf10, 1.6 (0.1–11) vs. 1.6 (0.58–20) pg/mL, *p* = 4.07 × 10^−1^ and P3H1, 7.8 (0–86) vs. 5.9 (0–586) pg/mL, *p* = 1.28 × 10^−1^ (double-sided Wilcoxon Rank Sum test). Cut off value, PPV, NPV of calculated AUC, sensitivity and sensitivity of each candidate biomarker are given in the online supplement (Supplementary Table 1). Although seven of the proteins differed significantly between patients and controls, they showed considerable variability. This indicated that on their own, none of the proteins would suffice as potential biomarkers for early diagnosis. This led us to test if a combination of all nine proteins separated patients and controls with high accuracy. For that purpose, we calculated AUC and *p* value as before (double-sided Wilcoxon Rank Sum test *p* = 1.62 × 10^−19^; Fig. 5). While the *p* value was highly significant, there was some overlap between patients and controls, resulting in a specificity of 92% and a sensitivity of 90%.

**Figure 6:**
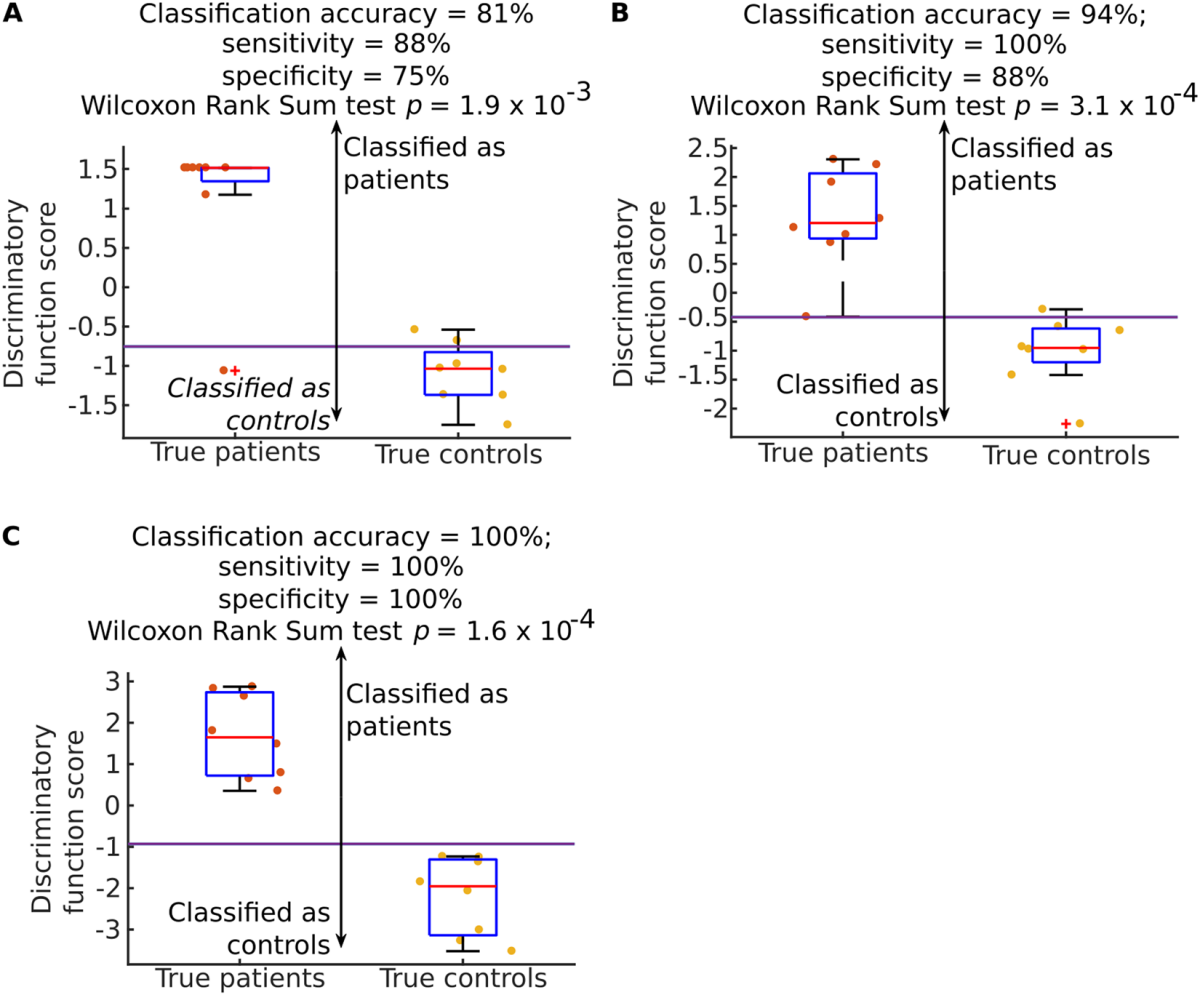
Classification accuracy of different combinations of two, three and four proteins in a test set of 8 patients and control samples. Boxplot presenting discriminatory function score based on the protein expression of combinations of two, three and four proteins in plasma samples of patients and controls in test group measured with ELISA. The protein combinations were: (**A**) TRIM28 and PLOD1, (**B**) TRIM28, PLOD1 and CEACAM5, C) TRIM28, PLOD1, CEACAM5 and P4HA1. Discriminatory function score was calculated as the sum of the concentrations of respective proteins. The discriminatory cut-off value is presented with the solid purple vertical line. The dark and bright orange dots denote values obtained for patients and controls in the test group respectively. The bars in the boxes represent median, 25^th^ and 75^th^ percentiles, while whiskers extend to ±2.7 σ. For the combination of two proteins we obtained best results when summing concentrations of TRIM28 and PLOD1 (sensitivity of 88%, specificity of 75%). For the combination of three proteins we yielded sensitivity of 100% and specificity of 88% (sum of TRIM28, PLOD1, and CEACAM5). However, best separation was possible when using four proteins, namely TRIM28, PLOD1, CEACAM5, and P4HA1 (both sensitivity and specificity of 100%).

This led us to test if there could be a more accurate combination of a smaller number of proteins. We started by testing all different combinations of the nine proteins in a training set consisting of randomly selected 72 patients and 72 controls. After that we tested the identified combinations in a test set consisting of the remaining 8 patients and 8 controls (Fig. 6).

## Discussion

A large percentage of patients with CRC are not detected in early disease stages. Since prognosis is highly dependent on disease stage, discovery of improved biomarkers for early diagnosis is a key priority. Currently, national screening programs mainly rely on detection of occult blood in stools. This increases the number of CRC patients that are diagnosed at early stages. However, because of limited specificity this results in many colonoscopies of healthy subjects. Another problem is limited compliance for colonoscopy, because it is perceived by many as difficult and painful. Painstaking research efforts during the last decades have resulted in many different biomarkers for early diagnosis. Ideally, these should be measurable in blood or stools, using routine clinical methods. Examples include measuring the FAR (fractional albumin rate) (FAR = 100 * Fibrinogen/Albumin), FPR (fibrinogen to prealbumin ratio) (FPR = Fibrinogen/pre-Albumin), and the neutrophil–lymphocyte ratio (NLR). Increased FAR, FPR and NLR have been reported in early CRC, as compared to healthy controls. Therefore, they could be implemented for early diagnostics^21^.

Alternatively, in large centers, more complex methods may have potential, particularly if many samples can be simultaneously analyzed. A promising example is cell-free DNA (cfDNA, which consist of DNA fragments from tumor cells), which may be detected in low quantities in serum or plasma^22^. Recently, analyses of methylation of the promoter of septin 9 have been described^11,12^. Other examples are microsatellite instability (MSI), which may serve as screening marker for Lynch syndrome^23^, and detection of mutated DNA in stools. A problem with this method is that only a fraction of stool DNA is human, while the remainder is bacterial^24^.

Antibody-based chips that measure multiple proteins in serum have also been proposed. Such chips exemplify how a large number of samples can be simultaneously and automatically analyzed in a short time period^25^. This method also highlights the observation that combinations of biomarkers may be more accurate than individual biomarkers ones^13^.

Here, we performed a meta-analysis of expression profiling and proteomic data from CRC tumors and AT. Our aims were to identify combinations of a limited number of proteins that could be measured in peripheral blood with routine methods, and test if our recently described classification algorithm could separate patients from controls, based on analyses of blood samples. The potential clinical benefit could be that using our algorithm such biomarkers could be used as a complement to screening, for subjects who have screened positively in order to optimize selection of those that are subjected to colonoscopy.

An advantage of the meta-analysis approach was that potential combinations could be validated and optimized in more than one material. Moreover, we could use another important resource, the Human Protein Atlas, to select proteins that could potentially be analysed in blood. This led to the identification of nine proteins, seven of which differed significantly in plasma concentrations between CRC patients and controls. Three of the seven proteins that differed significantly had already been identified as potential biomarkers, CEACAM5 (commonly known as CEA), LCN2 and TRIM28^16^.

In order to identify optimal combinations of the nine proteins we started by developing classifiers for all possible combinations of the nine proteins in a training set consisting of 90% of randomly selected patients and 90% of the controls. Next, we tested the most accurate classifiers for two, three and four proteins in the remaining 10% of patients and controls. We found that the combination of four proteins had a 100% sensitivity and specificity, namely TRIM28, PLOD1 and CEACAM5 and P4HA1.

Although the diagnostic potential of these proteins need confirmation in larger materials, which takes into account potential confounders like age, gender and ethnicity, their relevance is supported by their known and potential pathogenic roles: CEACAM5 may promote tumor development by acting as a cell adhesion molecule, and by regulating differentiation, apoptosis, and cell polarity. LCN2 is a pleiotropic protein, which depending on tissue and molecular context may either contribute to or oppose tumorigenesis^26^. Interestingly, TRIM28 is a transcriptional corepressor, with complex and context-dependent effects. For example, in a mouse model of liver cancer TRIM28 opposes tumorigenesis^27^. Of the remaining four proteins whose plasma concentrations differed significantly between patients and controls, PLOD1 has an important role in extracellular matrix formation^28^. This protein showed a highly significant increase in CRC patients. P4HA1 regulates collagen formation, and has been implicated in melanoma^29^. MAD1L1 has a potential role in driving aneuploidy, and genetic variants in this gene has been associated with CRC^30^. GNS has no clear oncogenic role, but mutations described in prostate cancer^31^.

Thus, the identified proteins had roles or genetic associations relevant to CRC. Moreover, network analysis of these nine proteins showed that they were highly interconnected and formed a network module, which regulated cell death and proliferation. Since it is known that proteins associated with the same disease tend to be interconnected and be functionally related, the high interconnectivity of the nine proteins supports their pathogenic and biomarker relevance^32^. Given that we started by meta-analysis of the more than 20,000 human transcripts and proteins, it is noteworthy that our biomarker prioritization algorithm identified nine proteins, that jointly were involved in the regulation of the same cellular function, which was potentially relevant to CRC pathogenesis.

From a practical clinical perspective, an advantage of the proposed proteins is that they can be measured with ELISA, which is a routine method that can be readily implemented in many laboratories.

A potentially important implication for future biomarker discovery could be to use the identified network module to find other types of biomarkers that interact with or regulate proteins in the module, such as genetic variants, microRNAs or DNA methylation. The algorithms used in this study could be applied to find increasingly accurate combinations of biomarkers^33^. While this may appear unrealistic today, the clinical need, decreasing analytical costs, improved software, and costs for colonoscopy may make such combinations realistic both from clinical and health economic perspectives. One possible scenario is that subjects who screen positive for blood in stools are examined with such combinations in order to optimize prioritization for further investigation with colonoscopy. Given the large number of individuals that do screen positively centralized laboratory and computational analyses may prove optimal in case increasingly complex methods are developed^34^.

It is of note that already today, hundreds of potential CRC biomarkers of different molecular types have been identified^14^.

Taken together, we propose that our analytical strategy and biomarkers may contribute to improved selection of subjects that are investigated with colonoscopy after screening positively. Further studies of larger cohorts are warranted to investigate this potential.

## Materials and Methods

### Protein prioritization using randomized elastic net

In order to rank-order proteins we analysed proteome profiling data of 22 CRC patients – paired samples taken from tumour and AT^15^. Data were downloaded from online Supplementary Information. For each identified protein fold change was calculated as average protein expression in colorectal tumour samples divided by average protein expression in AT. Differential expression was obtained from Supplementary Information^15^ (paired t-test). For biomarker prioritization using random elastic net we pre-selected those proteins that were: (a) differentially expressed (adjusted for multiple correction using procedure described by Storey *p* value < 0.01); (b) upregulated in CRC tumour samples (fold change more than 2); (c) predicted to be secreted according to Human Protein Atlas (https://www.proteinatlas.org, data downloaded in July 2017).

113 proteins fulfilling above criteria were rank-ordered by their predictive value using randomized elastic net. Randomized elastic net was implemented as a modification of randomized lasso as described by Meinshausen *et al*.^35^. Here, lasso was replaced with elastic net. For selected λ in cross-validation and for α = 0.5, we permuted data by adding random penalty factors for each predictor (protein) from the interval [1/α, 1]. Next, model coefficients were estimated (elastic net). We performed 100,000 permutations. Predictors with non-zero coefficients in at least one of the 100,000 permutations were selected. For downstream analyses proteins selected in at least 45% of permutations were chosen (n = 9 proteins; Supplementary Data 1).

### Sample classification

Based on the selected proteins we built a classifier with discriminative function being the sum of expression of all nine proteins. All zeros in proteomic data were replaced with NaN and treated as missing values (*nansum()* was used). To calculate Area Under ROC curve - AUC values - we used MATLAB function *perfcurve()*, having colorectal tumor (CRC) samples as positive group and adjacent tissue(AT) samples as negative group. In order to obtain significance values, we performed Wilcoxon Signed Rank test for paired samples and Wilcoxon Rank Sum test for non-paired samples on the scores calculated based on discriminative function score for both CRC and AT groups.

In order to test if selected proteins were better than by chance, we randomly selected nine upregulated proteins from the dataset and repeated calculation of AUC scores 10,000 times. Permutation *p* value was calculated by comparing random AUC values with the original one.

### Validation in independent datasets

To test selected nine proteins, we have repeated classification and classification tests as described above in two independent, publicly available, proteomics datasets: (1) consisting of 101 individuals (CPTAC), samples taken from tumour site (CRC) and adjacent tissue (AT). For classification we used 2 to the power of reported “Unshared Log Ratio” score, unpaired samples were removed from the analyses; (2) Consisting of four normal mucosa samples, four inflamed and eight early cancer samples (PXD005735). Additionally, we analysed if the transcriptome profiling of the nine genes encoding selected proteins could discriminate CRC and AT. We analysed the following datasets: (1) EGEOD-77955: 6 normal surface epithelium, 7 normal crypt epithelium, 17 CRC, 11 metastases, 17 adenoma samples (In total 19 subjects); (2) E-GEOD-41258: 54 normal colon tissues, 186 CRC, 49 polyp samples; (3) E-MTAB-3732, which aggregated and normalized microarray datasets from different studies of healthy and diseased colorectal tissues. This included 74 healthy colon tissues, CRC from three studies with 4, 288 and 52 patients, 30 adenomas, 4 familial hyperplastic polyposis and 47 ulcerative colitis.

### Discriminative score and clinical data

In the publically available dataset consisting of 100 individuals (CPTAC) we also tested if the discriminative score was influenced by sex, histological subtype, history of prior polyps, and race Wilcoxon signed rank test where samples were paired and Wilcoxon rank sum test otherwise between specific subgroups of samples.

### ELISA of plasma samples from patients with CRC and healthy controls

The study was reviewed and approved by the Regional Ethical Review Board in Linköping, Linköping, Sweden (98113 and 2013/271-31). All methods were performed in accordance with the relevant guidelines and regulations. All patients included in this study gave an informed written consent for utilization of their material in research.

80 CRC patients (40 females and 40 males, mean age of 71.8 years (range 34–89)) were recruited from south-eastern Sweden who had undergone surgical resections for primary CRC at the Department of Surgery, Division of Surgical Care, Region Jönköping County, Jönköping, Sweden. The CRC patients had tumours localized in colon (n = 37) or rectum (n = 43) with TNM stages I-IV (I = 13, II = 34, III = 29 and IV = 4). The control group consisted of 80 healthy blood donors (40 females and 40 males, mean age of 55.9 years (range 33–67)) with no known history of CRC and from the same geographical region as the cancer patients. Venous blood samples were collected during surgery and centrifuged within 1 hour to separate plasma and blood cells. Plasma samples were stored at −80 °C in the Biobank of Laboratory Services, registration number 868, Region Jönköping County, Jönköping, Sweden until analysis. Plasma levels of the nine potential biomarkers were analysed using commercial Enzyme-Linked Immunosorbent Assays (ELISAs) according to the manufacturer’s instructions; C12orf10 (MyBiosource, Inc., San Diego, CA, United States), CEACAM5 (LifeSpan BioSciences, Inc., Seattle, WA, United States), GNS (MyBiosource, Inc.), LCN2 (Aviva Systems Biology Corp., San Diego, CA, United States), MAD1L1 (Abbexa Ltd., Cambridge, United Kingdom), P3H1 (Abbexa Ltd.), P4HA1 (Signalway Antibody LLC, Baltimore, MD, United States), PLOD1 (Aviva Systems Biology Corp.), and TRIM28 (MyBiosource, Inc.). Protein levels of the nine potential biomarkers were determined using the Sunrise Tecan Microplate reader (Tecan Austria GmbH, Salzburg, Austria) along with the Magellan 7.x 2010 software (Tecan Austria GmbH). Protein levels of C12orf10, GNS, LCN2, MAD1L1, P4HA1, PLOD1 and TRIM28 were expressed as nanograms per millilitre (ng/mL). Protein levels of CEACAM5 and P3H1 were expressed as picograms per millilitre (pg/mL). In case protein values were out of ELISA kit detection limit for calculations we assumed highest ELISA kit detection limit or a value of 0.

### Network analysis of the identified nine proteins

We used the Ingenuity Pathway Analysis (IPA) software (Qiagen, Hilden, Germany) to test if the nine proteins were part of the same network module.

Sub-network formed by IPA was done as follows: First all genes having direct or indirect interactions with the nine proteins serve as “seeds” to generate networks. Such focus genes are combined into networks to maximize their specific interconnectivity (the connectivity between focus genes in comparison to the number of their interactions with other genes within the IPA global network). Additional genes from the IPA global network are added in order to connect smaller networks formed by focus genes. Finally resulting networks are scored based on the number of focus genes they contain - the higher score the lower probability of finding this number of focus genes within given network by random chance.

### Sample separation to training and testing sets

For each of the nine individual proteins we tested if they differed significantly between patients and controls using the double- sided Wilcoxon Rank Sum test. Next, we summed the expression of all nine proteins in order to obtain a discriminatory function score and calculated AUC (as before we used MATLAB *perfcurve()* function for that purpose). Even though based on the tissue samples (proteome profiling) all nine proteins should be upregulated in patient samples however in the plasma samples some of the proteins were in fact down regulated in patients compared to controls. Therefore, concentrations of those proteins was subtracted from the discriminatory function score instead of being summed. *P* value was calculated using double-sided Wilcoxon Rank Sum test. Next, we aimed to reduce the number of proteins necessary for the classifier to perform well. For these analyses, ten percent of patient samples (n = 8) and ten percent of control samples (n = 8) were randomly selected as test group and excluded from the initial analyses, in which we tried different combinations of the measured proteins. The optimal operating point (discriminatory cutoff value) was selected using MATLAB function *perfcurve()*. This is based on moving a straight line from the point (0,1) with a slope defined as $S=\frac{Cost(FP)-Cost\,ADDIN\,EN.CITE\,ADDIN\,EN.CITE.DATA\,2}{Cost\,ADDIN\,EN.CITE\,ADDIN\,EN.CITE.DATA\,21-Cost\,ADDIN\,EN.CITE\,ADDIN\,EN.CITE.DATA\,2}\cdot \frac{TN+FP}{TP+FN}$ that crosses ROC curve. Here TP, FP, TN, and FN are the true positives, false positives, true negatives and false negatives respectively, and classification cost is denoted as *Cost()*. Since it is most important to reduce probability of false negatives (classification of patients as healthy controls) we assumed zero cost for classification of TF and TP, 0.8 cost for misclassification of positive class, and 0.2 for misclassification of negative class.

### Classification of the test group

Finally, using the classifier we constructed as described above, we classified the left-out samples of patients and controls, calculated accuracy, sensitivity and specificity, and *p* value (*p* value was calculated based on the discriminatory function score obtained for the left-out samples between patients and controls using double-sided Wilcoxon Rank Sum test).

## Supplementary information

Supplementary Information

Supplementary Dataset 1

Supplementary Dataset 2

## Data Availability

The transcriptomic and proteomic datasets analysed in the current study were derived from the public databases described above.
