# Supplementary material for: An algorithm-based meta-analysis of genome- and proteome-wide data identifies a combination of potential plasma biomarkers for colorectal cancer
Source: Sci Rep. 2019 Oct 30;9:15575. doi: 10.1038/s41598-019-51999-9 (PMC6821706; doi:10.1038/s41598-019-51999-9)
Supplement: Supplementary file 1 — Supplementary Information [file 41598_2019_51999_MOESM1_ESM.pdf]

An algorithm-based meta-analysis of genome- and proteome-wide data identifies a combination of potential plasma biomarkers for colorectal cancer

Danuta R. Gawel[1*], Eun Jung Lee[1,2], Xinxiu Li[1], Sandra Lilja[1], Andreas Matussek[3,4,5], Samuel Schäfer[1], Renate Slind Olsen[6,7], Margaretha Stenmarker[8], Huan Zhang[1*§], Mikael Benson[1§]

Affiliation

[1]Centre for Personalized Medicine, Linköping University, Linköping, Sweden.

[2]Department of Otorhinolaryngology, Yonsei University College of Medicine, Seoul, Korea

[3]Laboratory Medicine, Division of Psychiatrics & Rehabilitation & Diagnostics, Region Jönköping County, Jönköping, Sweden.

[4]Division of Clinical Microbiology, Department of Laboratory Medicine, Karolinska Institute, Karolinska University Hospital Huddinge, Stockholm, Sweden.

[5]Karolinska University Laboratory, Karolinska University Hospital, Solna, Sweden.

[6]Pathology Laboratory, Division of Psychiatrics & Rehabilitation & Diagnostics, Region Jönköping County, Jönköping, Sweden.

[7]Center for Translational Microbiome Research, Department of Microbiology, Tumor and Cell Biology, Karolinska Institute, Stockholm, Sweden.

[8]Futurum, Region Jönköping County, and Department of Clinical and Experimental Medicine, Linköping University, Linköping, Sweden.

§These authors have equal contributions.

*__Correspondence:__ danuta.r.gawel@gmail.com (D.R.G.) huan.zhang@liu.se (H.Z.)

Supplementary Figures

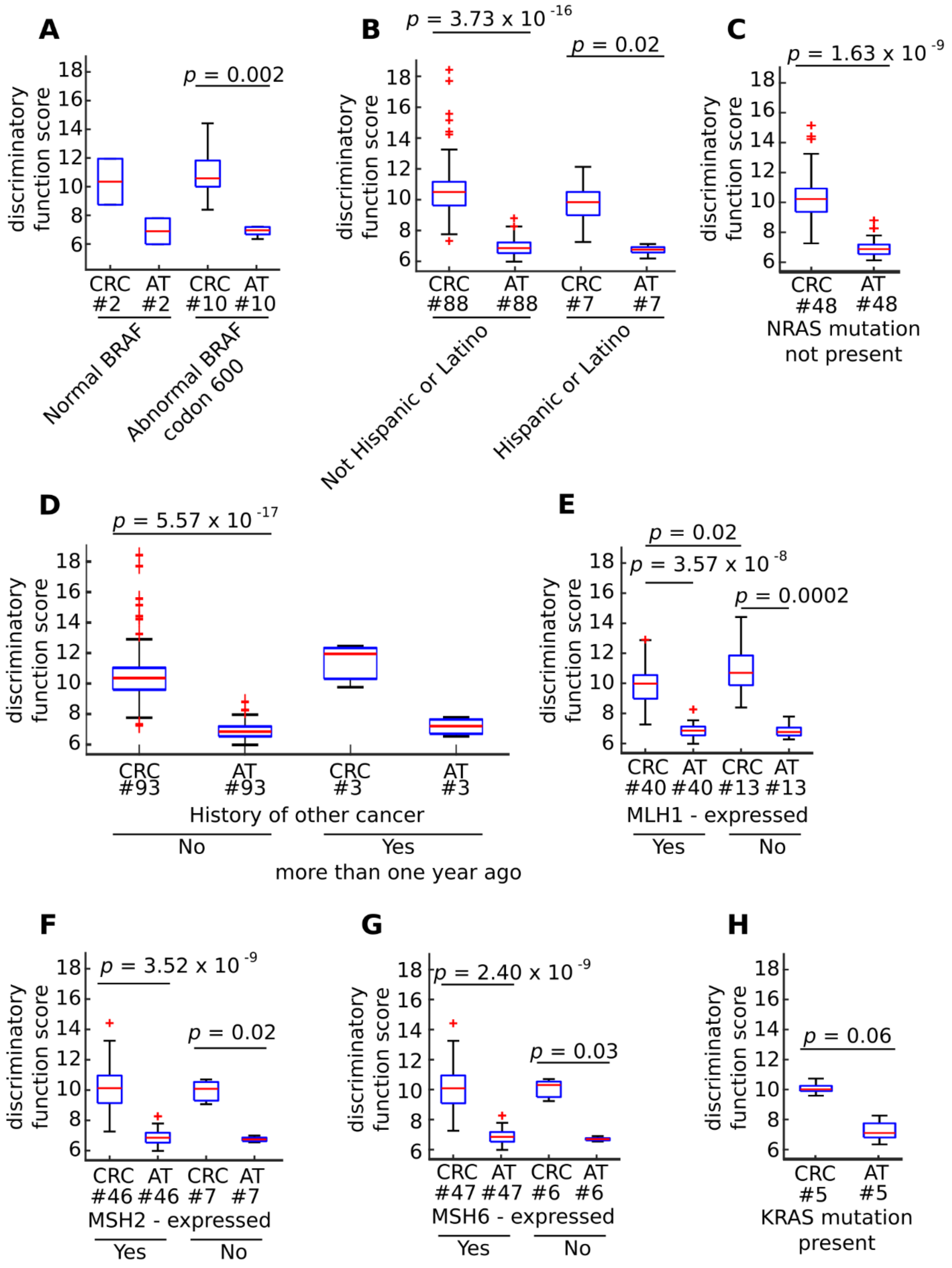

Supplementary Figure 1. Classification accuracy of colorectal cancer tumor samples (CRC) and adjacent tissue (AT). Boxplot presenting discriminatory function score for CRC and AT groups (sum of the 2 to the power of Unshared Log Ratio scores reported in the study for selected proteins: PLOD1, P4HA1, LCN2, GNS, C12orf10, P3H1, TRIM28, CEACAM5, MAD1L1). Significance *p* value was calculated using double-sided Wilcoxon Signed Rank test for paired samples and Wilcoxon Rank Sum test for unpaired samples. The bars in the boxes represent median, 25th and 75th percentiles, while whiskers extend to ± 2.7σ. Numbers below the boxplots denote number of observations per category. Discrimination between CRC and AT samples divided by (**A**) BRAF gene analyses results; (**B**) Ethnicity; (**C**) Presence of NKRAS mutation (only one sample with no NKRAS mutation is

reported in the study, see Supplementary Data 2) (**D**) History of other cancers; (**E**) MLH1 expression; (**F**) MSH2 expression; (**G**) MSH6 expression; (**H**) Presence of KRAS mutation (Only one sample is reported in the study with no KRAS mutation, see Supplementary Data 2).

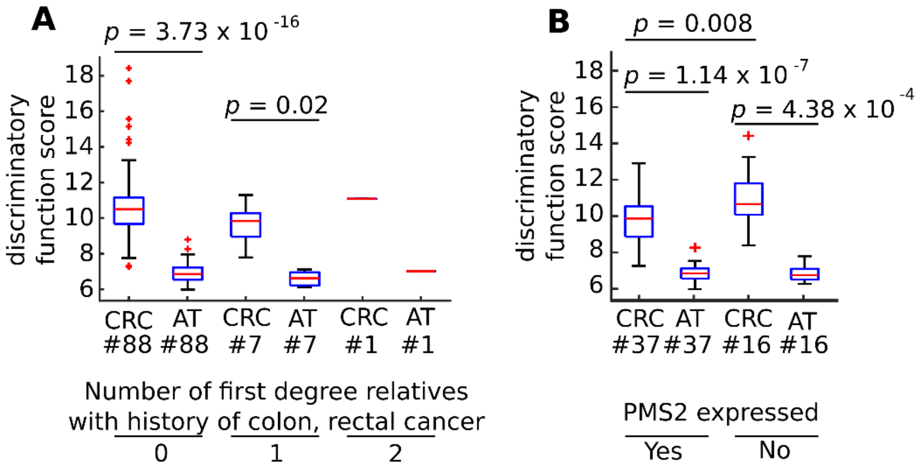

Supplementary Figure 2. Classification accuracy of colorectal cancer tumor samples (CRC) and adjacent tissue (AT). Boxplot presenting discriminatory function score for CRC and AT groups (sum of the 2 to the power of Unshared Log Ratio scores reported in the study for selected proteins: PLOD1, P4HA1, LCN2, GNS, C12orf10, P3H1, TRIM28, CEACAM5, MAD1L1). Significance *p* value was calculated using double-sided Wilcoxon Signed Rank test for paired samples and Wilcoxon Rank Sum test for unpaired samples. The bars in the boxes represent median, 25th and 75th percentiles, while whiskers extend to ± 2.7σ. Numbers below the boxplots denote number of observations per category. Discrimination between CRC and AT samples divided by (**A**) Number of first degree relatives with history of colon, rectal cancer; (**B**) PMS2 expression.

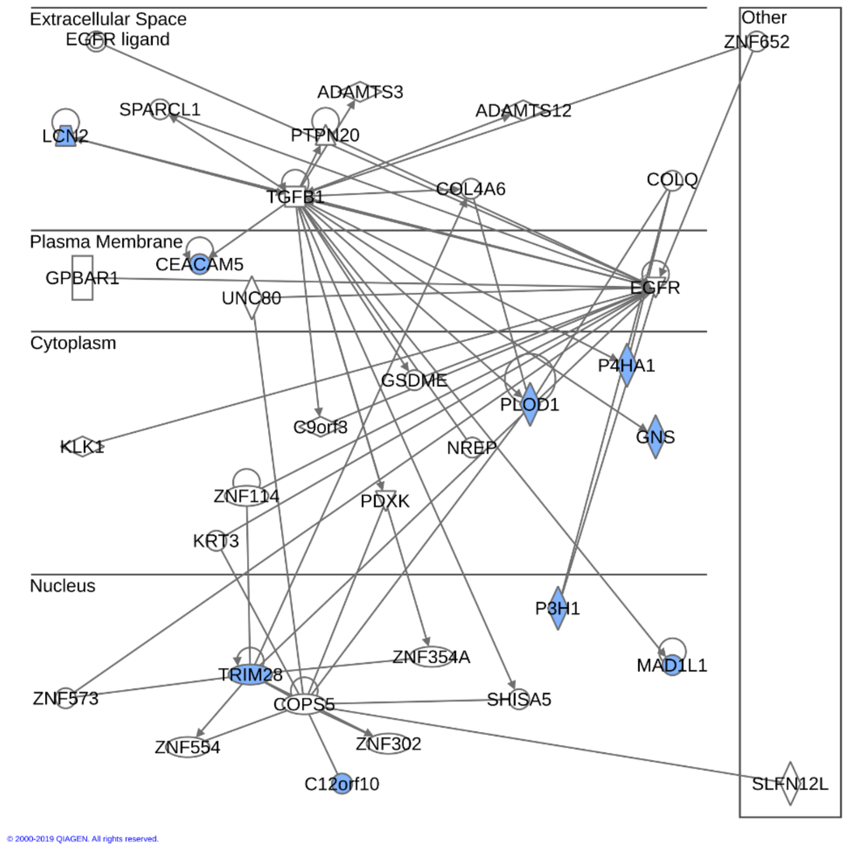

© 2000-2019 QIAGEN. All rights reserved.

Supplementary Figure 3. Ingenuity Pathway Analysis shows that the nine proteins (blue) are highly interconnected and form a network module that regulates cell death and proliferation. In order to test the pathogenic relevance of the nine biomarkers we performed Ingenuity Pathway Analysis, as previously described[1]. Briefly, the background is that proteins that are associated with the same disease tend to be functionally related and interact, forming network modules [1]. Thus, if the proteins did interact, this would support their pathogenic and biomarker relevance. Indeed, we found that the nine proteins did form a network module, which had an overriding function, namely regulating cell death and proliferation.

	**Selected cutoff**	**AUC**	**PPV**	**NPV**	**sensitivity**	**specificity**
**LCN2**	-3,57	0,52	0,50	--	1,00	0
**PLOD1**	0,22	0,93	1,00	0,89	0,88	1,00
**MAD1L1**	-3,19	0,56	0,53	1,00	1,00	0,13
**P3H1**	-0,43	0,55	0,50	--	1,00	0
**CEACAM5**	-0,79	0,63	0,50	--	1,00	0
**P4HA1**	-1,85	0,68	0,62	1,00	1,00	0,25
**C12orf10**	-3,40	0,56	0,50	--	1,00	0
**GNS**	-0,05	0,84	0,75	0,75	0,75	0,75
**TRIM28**	0,30	0,98	0,88	0,88	0,88	0,88

Supplementary Table 1. The nine individual biomarkers were iundividually tersted as classifiers for CRC. Briefly we have created classifiers using 90% of the patient samples (calculate cut-off values) and we have tested the classifier based on the remaining 10% of the samples. We have obtained following cut-off values, positive and negative predictive values, and AUCs. Missing negative predictive values were obtained for classifiers where all samples were classified as "positive" i.e. patient samples.

Description of Additional Supplementary Files

Supplementary Data 1. Randomized elastic net results. 113 ordered by their predictive value (randomized elastic net frequency).

Supplementary Data 2. Covariate impact on the discriminatory function score. We tested if discriminatory function score differs depending on covariates. P values were calculated using Wilcoxon Signed test where applicable or Wilcoxon Rank Sum test. All p values together with the number of samples per group are reported in separate excel sheet.

References

1 Gustafsson, M. *et al.* A validated gene regulatory network and GWAS identifies early regulators of T cell-associated diseases. *Science Translational Medicine* **7** (2015).
